# European research Priorities for Osteopathic Care (PROCare): a sequential exploratory investigation and survey

**DOI:** 10.1136/bmjopen-2025-100757

**Published:** 2025-10-16

**Authors:** Paul Vaucher, Dawn Carnes, David Hohenschurz-Schmidt, Oliver Thomson, Steven Vogel, Chiara Arienti, Philip Bright, Gerard Alvarez Bustins, Jorge Esteves, Nuno Koch Esteves, Carol Fawkes, Sandra Rinne, Sonia Roura, Loïc Treffel, Agathe Wagner, Jerry Draper-Rodi

**Affiliations:** 1 Centre for Osteopathic Research and Leadership (CORaL), Health Sciences University UCO School of Osteopathy, London, UK; 2 Department of Health, Swiss Distance University of Applied Sciences, Zürich, Switzerland; 3 Department of Health, University of Applied Sciences Western Switzerland (HES-SO), Fribourg, Switzerland; 4 Foundation COME Collaboration, Pescara, Italy; 5 Imperial Clinical Trials Unit, School of Public Health, Imperial College London, London, UK; 6 Faculty of Public Health, UTS ARCCIM, Sydney, New South Wales, Australia; 7 Clinical Epidemiology Research Center (CERC), Department of Biomedical Sciences, Humanitas University, Milan, Italy; 8 Iberoamerican Cochrane Centre, Biomedical Research Institute Sant Pau, Barcelona, Spain; 9 Department of Physical Therapy, Blanquerna Universitat Ramon Llull Facultat de Ciencies de la Salut, Barcelona, Spain; 10 Escola Superior de Saúde Atlântica, Barcarena, Portugal; 11 School of Health Sciences, Skin Sensing Research Group, Thermosenselab, University of Southampton, Southampton, UK; 12 National Council for Osteopathic Research, London, UK; 13 Metropolia University of Applied Sciences, Helsinki, Finland; 14 Medical Department, Universitat Autonoma de Barcelona, Barcelona, Spain; 15 Institut Toulousain d’Ostéopathie, Toulouse, France; 16 Centre Européen d’Enseignement supérieur d’Ostéopathie, Paris, France

**Keywords:** Physical Therapy Modalities, Surveys and Questionnaires, Research Design, Health, Community Participation

## Abstract

**Abstract:**

**Objectives:**

The aim of this study is to identify and analyse research priorities across the osteopathic profession internationally, to determine how different interested parties conceptualise research importance and to examine how contextual factors influence research prioritisation.

**Design:**

A mixed methods sequential exploratory design combining an umbrella review, a thematic analysis, an expert consensus agreement and an international cross-sectional survey was used to define, validate and evaluate research priorities.

**Setting:**

An international online survey, available in nine languages, was distributed through professional osteopathic organisations and network worldwide, a patient representative organisation and social media.

**Participants:**

2229 respondents including patients (7.4%), practitioners (42.1%), students (17.4%), educators (13.5%), researchers (5.0%) and policy makers (4.3%) from across 42 countries.

**Primary and secondary outcome measures:**

Primary outcomes were interested party’s conceptualisation of research importance and validation of the priorities in Research for Osteopathic Care (PROCare) framework. Secondary outcomes included current research priorities across interested parties groups and influence of contextual factors on prioritisation.

**Results:**

Three distinct approaches to priority-setting emerged: conservative (42.9%), sceptic (20.2%) and enthusiast (36.9%). Organising research priorities as a construct built from domains and subdomains was shown to be internally valid (Cronbach’s α=0.911). ‘Patient safety’ (nominated by 82% of relevant countries) and ‘physical activities and mobility’ (51.0%) were the most prioritised subdomains. ‘Digital health’ ranked lowest (28th of 28 subdomains). Significant geographic variations were observed mainly for the overall importance to most research domains. Strong consensus emerged around core priorities including patient safety, physical activity promotion and understanding treatment mechanisms.

**Conclusions:**

The PROCare framework provides a validated structure for evaluating osteopathic research priorities across diverse interested parties. While geographic variations exist in priority emphasis, fundamental agreement on key research domains suggests potential for internationally coordinated research strategies. Future work should focus on developing mechanisms to ensure balanced representation of conservative, sceptic and enthusiast perspectives in research planning.

**STRENGTHS AND LIMITATIONS OF THIS STUDY**

- Largest international survey to date of osteopathic research priorities (N=2229) providing robust statistical power for subgroup analyses.
- A novel sequential exploratory approach was used to develop, validate and evaluate research priorities.
- Convenience sampling through professional networks may have introduced selection bias towards research-engaged participants.
- Variable response rates between countries (15–568 per 1000 osteopaths) may limit generalisability in some regions.
- Patient representation primarily from the UK (25.8%) may not reflect international patient perspectives.

## Introduction

 Research priority-setting in healthcare requires systematic engagement with interested parties to ensure that investigations address clinically relevant questions and optimise resource allocation. Within osteopathy, identifying research priorities is particularly crucial given the profession’s varied scope of practice internationally and ongoing evolution of evidence-based approaches.^1 2^ Osteopathic physicians in the USA are fully licensed medical doctors with additional training in osteopathic manipulative treatment, while osteopaths trained elsewhere may not be licensed physicians and often have a more limited scope of practice focused on manual therapy.^3^ In Europe, even if unified standards for education have been formulated,^4^ osteopathic care and its integration into health systems varies considerably between countries ranging from countries with no regulation (eg, Germany, the Netherlands, Spain), passing by countries with regulation as complementary medicine without medical scope (eg, France, Italy), to those practising with regulated university degree primary care status (eg, the UK, Switzerland).^5^ Identifying priorities for research within this scope of practice is crucial for directing resources towards areas that have the potential to make a significant impact on care provision within national health systems.^6^

Patients and osteopathic practitioners possess first-hand knowledge of the daily challenges faced in clinical practice that can be very valuable when setting research priorities.^7 8^ They bring a unique perspective, grounded in their lived experiences with healthcare conditions, treatments and outcomes.^9^ Patient participation has therefore been increasingly stressed in healthcare biomedical research.^710^,^12^ Traditionally, research priorities in healthcare have largely been determined by researchers, funding agencies and policymakers, with only 9% of research priority documents actively involving interested parties.^13^ For example, when setting research priorities, WHO documents rely mostly on expert researchers’ opinions (86%) and literature reviews (52%).^14^ While this type of initiative brings valuable expertise and insights, there has been a realisation that research agendas should also incorporate the perspectives of those who directly experience and deliver healthcare services.^13 15^ Their involvement in setting research priorities increases the likelihood that studies address real-world issues and have direct relevance to patient care.^16^

In 2014, a Delphi consensus study on osteopathic research identified the following priorities: clinical effectiveness, patient safety and risks related to treatment, role and scope of osteopathic practice and outcomes of osteopathic treatment.^17^ As the authors of that study noted, at the time, there had already been considerable research done on most of these topics, which apparently the respondents were unaware of, suggesting a knowledge transfer barrier/problem. Osteopaths, like physiotherapists,^18^ have positive attitudes towards evidence-based practice (EBP), but struggle to engage with research due to limited skills and practice critically appraising and interpreting research sources.^19^,^23^ In the Delphi consensus study,^17^ reaching a consensus on ‘research importance’ was difficult to achieve as different participants attributed different meaning to the concept of priority shaped by their views, opinions and values for societal needs, scientific novelty and resource allocation. Investigating research priorities has been improved by conceptualising and defining the notion of priority^24^ and framing research themes as principal research domains, subdomains and topics.^25^

In summary, existing osteopathic research priority frameworks were not sufficiently informed by practitioners, students, educators and the wider public, arguably limiting their impact. Further, osteopathy is a diverse profession with considerable regional variability in practice, education and regulation across Europe, which has not been accounted for in previous research priorities.^5^ For these reasons, it is timely to update and investigate public and practitioners’ views on research priorities in osteopathic care and see how these might differ between interested parties, between people with different values according to research priorities and between countries.

The priorities in research for osteopathic Care (PROCare) project aimed to develop and validate an evidence-based framework for investigating research priorities, using osteopathic care as an exemplar. The study addressed four interconnected research questions.

### Primary research questions

1. How do different interested parties conceptualise and evaluate research importance in osteopathic care? (Objective 1)
2. To what extent can research priorities be systematically categorised using the PROCare framework? (Objective 2)

### Secondary research questions

1. What are the current research priorities across different interested parties? (Objective 3)
2. How do contextual factors influence research prioritisation? (Objective 4)

## Methods

### Design

The PROCare framework was developed using a sequential exploratory mixed-methods design, where findings from each phase informed the next. The umbrella review generated a master list of potential priorities, which was thematically analysed to produce a taxonomy of domains, subdomains and topics. This taxonomy was refined through expert validation, resulting in a conceptual framework that guided the construction of the survey. The draft survey was then tested, validated and culturally adapted before being deployed between August and October 2023 to quantitatively assess the perceived importance of research priorities among practitioners, educators, researchers, policymakers and from the public including patients.

### Studied population

A comprehensive purposive sampling strategy targeted key interested parties through multiple channels: (1) Professional organisations: All member associations of Osteopathy Europe (OE) received standardised recruitment materials for distribution to their members; (2) Research networks: The Centre for Osteopathic Medicine Collaboration and National Council for Osteopathic Research disseminated invitations through their established networks; (3) Educational institutions: Accredited osteopathic educational institutions in participating countries shared recruitment materials with faculty and students; (4) Patient groups: Established patient participation groups in the UK provided access to service user perspectives; and (5) Social media: Standardised recruitment posts were shared through professional osteopathic networks. Each participating organisation distributed two waves of invitation emails between August and October 2023 using a common survey link. No incentives were offered for participation.

The survey was open for 45 days, with associations sending invitation emails and reminders after 2–4 weeks. The survey was designed to be run in countries represented in OE. This included Belgium, Denmark, Finland, France, Germany, Ireland, Italy, the Netherlands, Norway, Portugal, Spain, Sweden, UK and the OE-affiliated Brazil and Canada. Switzerland, as a previous OE member, was also included. Responders had to be adults.

### Questionnaire development and testing

The PROCare framework was developed through a systematic five-phase process: (1) literature synthesis; (2) thematic analysis; (3) expert validation; (4) framework finalisation and (5) survey questionnaire development and validation. Materials used during these phases are made available on Zenodo^26^ (https://doi.org/10.5281/zenodo.8303132).

#### Literature synthesis: umbrella review

A master list of priorities was determined by the umbrella literature review identifying published lists of research priorities and topics published between 1998 and 2023. These were reviewed by a panel of experts and patient representatives, and a list of items for inclusion in the survey was agreed.^27^

PubMed was searched for publications from 1998 to 2023 using the key terms "research priorities", "Delphi" or "Survey", "Primary care" or "General practice" or "Chiropractic" or "Physiotherapy" or "Osteopathic" or "Sports medicine" OR "Patients" or "Stakeholders", by a single researcher. Inclusion criteria were publication after 1997, the survey had to concern priorities in research in public health, primary care, physiotherapy, osteopathic or chiropractic care, or sports medicine, and the study had to investigate priorities generally rather than specifically for a condition. On 28 May 2023, PubMed listed 136 articles of which 12 were retained.^1728^,^38^ Forward and backward tracking identified an additional four studies.^2539^,^41^

#### Thematic analysis: taxonomy

From these 16 studies, data were extracted on the methods used to define the master list of priorities, on the surveyed population and on the categorisation used for listing research domains and subdomains within each study. Content interpretative thematic analysis^42^ was then used to identify underlying taxonomy for organising research priorities. Data analysis was done on Taguette 1.4.1.^43^ Details of these study and the thematic analysis can be found in the File no 6 within the shared data^26^ (https://doi.org/10.5281/zenodo.8303132).

**Figure 1:**
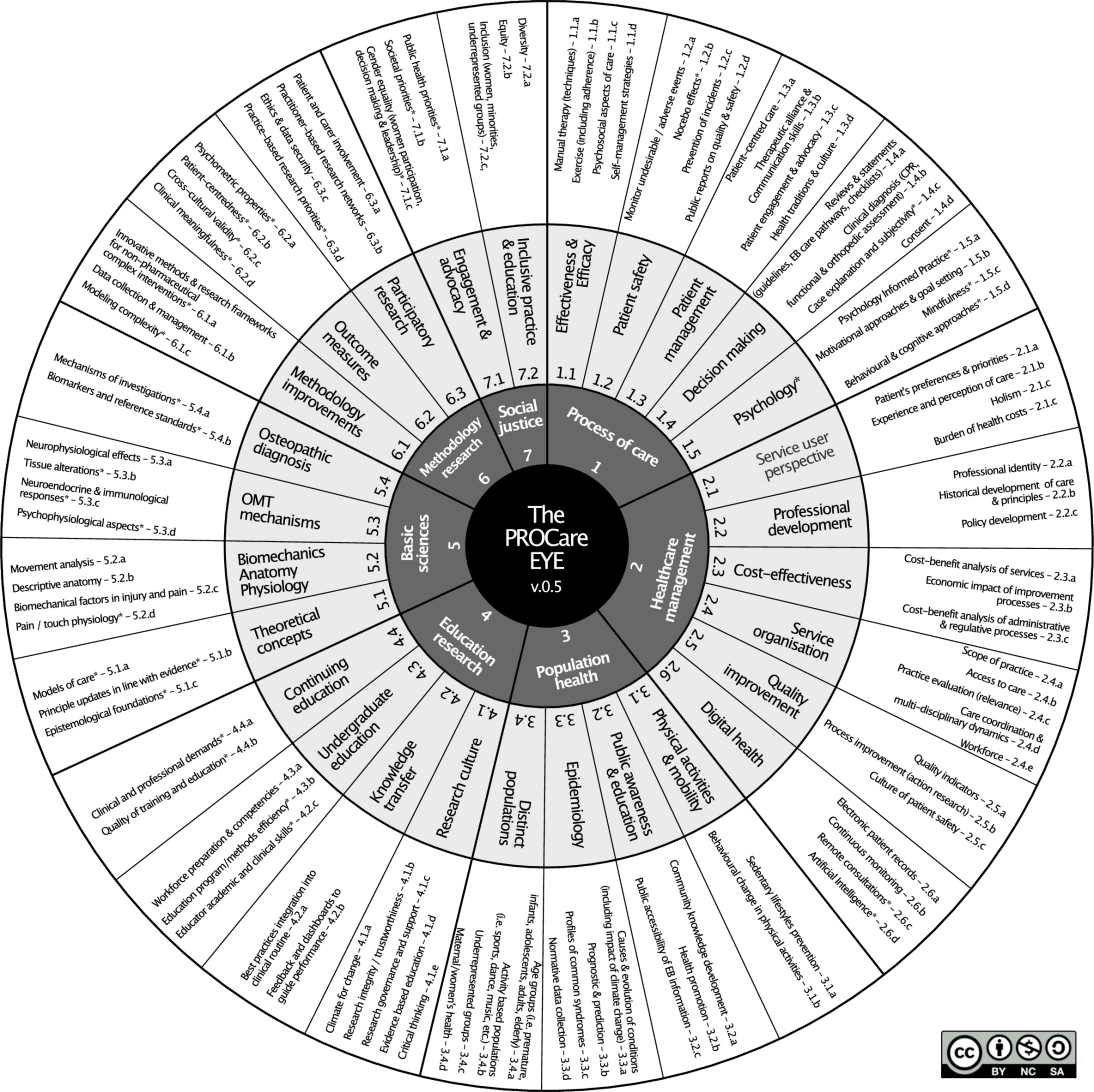
The Priorities in Research for Osteopathic Care (PROCare) Eye. A hierarchical model for classifying osteopathic research priorities comprising seven principal domains (inner circle), 28 subdomains (middle circle) and 96 research topics (outer circle). Asterisks (*) denote topics validated through expert consensus but not derived from literature review. The circular visualisation emphasises the interconnected nature of research priorities while maintaining clear categorical distinctions. Domain clustering reflects thematic relationships identified through principal component analysis. CPR, clinical predictive rule; EB, evidence based; OMT, osteopathic manual treatment.

Within the literature, there seemed to be two overlapping systems of classification for health research priorities: one was person/service related, the other was health condition/disease related. Given that osteopathic care is claimed to be person-centred rather than disease centred,^44^,^47^ we chose to focus on the first system. This made it possible to label and categorise 246 known priorities into 7 principal research domains, 28 subdomains and 96 research topics. Research priorities were summarised into a model called the PROCare Eye (figure 1).

#### Expert validation

A first panel of five experienced academic researchers in the field of osteopathic care (ie, DC, JD-R, DH-S, OT and SV) was invited to assess construct and content validity, by reviewing and improving the labelling and classification. If third-level topics were missing from the qualitative analysis, semantic associations were searched using OpenAI’s (2023) ChatGPT-3.5 (May 3 version) (large language model) and were used as suggestions for experts to clarify. Then, the entire model was presented to 19 osteopathic researchers, including the 5 from the first panel, during a 2-day workshop on the theme of research priorities in London (19–20 July 2023). The group refined the framework and added a seventh ‘principal research domain’ and finalised the taxonomy.

#### Framework finalisation

Under the oversight of the 19 osteopathic experts from the previous phase, a survey was then constructed from the model and comprised five main sections:

1. Principal research domain priority assessment: Participants were presented with a list of subdomains derived from the literature review and were asked to rate the importance of each subdomain on a 5-step Likert scale, ranging from −2 (not important at all) to 2 (very important). Participants were asked to rate the importance of each of the seven principal research domains: process of care, healthcare management, population health, education, basic science, methodology and social justice.
2. Research subdomain priority assessment: Using the same method, participants were presented with a list of subdomains derived from the literature review and were asked to rate the importance of each subdomain within each of the six principal research domains.
3. Topic priorities and open-ended questions: This section aimed to capture nuanced perspectives and emerging themes that may not have been covered in the umbrella review. Participants were asked to select three relevant topics within each principal research domain and add any other suggestions.
4. Assessing criteria used to set priorities: Participants were asked to report what importance they assigned to different criteria when expressing their views on research priorities.
5. Demographic information: Participants were asked to provide demographic details, including age, gender, country in which they are most exposed to osteopathic care and feelings of belonging to different representation groups (patients, practitioners, policymakers, educators, researchers).

#### Survey questionnaire development and validation

The final version included 25 questions that required approximately 15 min to answer. Content validity, face validity, cognitive testing and translation and cross-cultural adaptation were carried out on the questionnaire: 15 new osteopaths with links to research, and 15 English speaking patient representatives were invited to go through the survey and assess comprehensibility, completeness, coherency, representativeness and applicability of each section. Questions were adapted from their comments and tested using a ‘think-aloud’ approach with three general public representatives that were naïve to healthcare jargon. The final validated version was translated from English to eight other languages using Deepl.com and improved by native speakers with experience in osteopathic research. The final questionnaire was made available in Dutch, Finnish, French, German, Italian, Portuguese, Spanish and Swedish. No back translations were performed. Construct validity of the PROCare Eye was statistically assessed using survey responses and principal component analysis (PCA).

### Monitoring and maintaining recruitment rates

Weekly progress reports were shared with the managing and steering committee throughout the period of data collection (August to September 2023). One reminder email to complete the survey was sent to all registered osteopaths through their professional association. A trilingual hot-desk support, answering concerns and questions, was made available using emails with responses sent within 24 hours. Responders were invited to provide feedback, comments, complaints or report difficulties to the research team.

### Defining interested party status

Self-report of lived experience was used to categorise interested parties into specific groups: patient, practitioner, student, educator, researcher or policy maker. Self-representation of group identity was therefore based on an individual analysis of personal traits based on experience and legitimacy to reflect on collective welfare for that group.^48^ Responders were asked ‘To what extent would you consider yourself as an expert in representing the following groups?’ using a 5-point Likert type scale (Not at all|A little|Somewhat|Considerably|Totally). If equal value were attributed to two or more group representation, responders were placed in the first group using the following order of priority: policy maker, researcher, educator, student, practitioner and patient.

### Defining profiles for setting priorities

The selection of research priority topics is dependent on an individual’s values and opinions about research.^24^ The aim of the study was to focus on research that would lead to ‘the most health benefits to the population that it serves within the budget constraint and while respecting equity considerations’. Responders were therefore asked to indicate the level of importance they attributed to research priorities in terms of maximising the benefits people receive from osteopathic care. At the end of the survey, they were asked about other values they might have considered. These were personal values and beliefs, expectations on overall reduction of burden from conditions/diseases, potential impact of change on clinical practice, valuing and promoting the profession, societal priorities and urgencies and funding opportunities. Based on the importance accorded to these values [−2; 2], latent class analysis tested whether ‘research importance’ was interpreted and perceived as a single common concept by all responders.

### Country

Responders were asked to choose a single country that represented the one in which they had the most osteopathic experience. The minimum number of responders from a single country that were required to provide stratified results for this country was set arbitrarily at n=50. Countries with fewer than 50 participants were grouped as ‘other’.

### Data management and analysis

The survey was administered online using a secure survey platform (Qualtrics, V.8.23, Provo, Utah, USA). Participants were given access to the survey through a common link. No identification was required nor collected, including IP addresses. Missing data were handled using listwise deletion for incomplete responses following sensitivity analysis confirming minimal impact on results. The duration of survey completion was analysed, and those completed in less than 2 min were discarded to avoid bot automated completion. All questions had the option ‘prefer not to answer’ to avoid any missing data. Answering all questions was compulsory to move forward in the survey.

Qualitative data from the open-ended questions were analysed using thematic analysis.^49^ Emerging domains, subdomains or topics that were not included in the PROCare Eye were identified and agreed on by the research team (ie, DC, JD-R, DH-S, OT, PV and SV).

The statistical plan was written and validated prior to any data analysis. Latent class analysis was conducted using maximum likelihood estimation to identify distinct profiles in priority-setting approaches. Model selection compared solutions with two to seven classes using Bayesian information criterion (BIC) as the primary fit index, supplemented by Akaike information criterion when BIC showed unstable fluctuation. The optimal solution was determined by lowest BIC value combined with theoretical interpretability of the resulting classes.^50^ PCA and confirmatory factor analysis were used to validate the construct of the PROCare Eye. The analysis was done entering all domains and subdomains within the same model. Bartlett’s test for sphericity was computed before proceeding to the PCA and the measure of Kaiser-Meyer-Olkin (KMO) sampling adequacy. Horn’s parallel analysis was then used to test whether the construct of domains or subdomains was unique or not. Finally, a VARIMAX-rotated component analysis of the retained factors was done to see which domains and subdomains were grouped together. KMO, Cronbach’s α and adjusted uniqueness were used to quantify the internal validity of the entire construct. Confirmatory factor analysis was then used to test whether the measures of the notion of ‘research importance’ were consistent with the proposed understanding of the nature of that construct. The latent variable was the overall feeling of importance for research in osteopathic care, and the measures were scores attributed to importance for domains and subdomains. Listwise deletion was used for missing data. Model fit was evaluated using absolute fit indices and relative fit indices.

Description of priorities was made by averaging the scores of importance of principle research domains and subdomains across responders, and by reporting the prevalence of those choosing topics as priorities within each domain.

Scores for domains were computed by adding those attributed to subdomains and dividing by the number of subdomains within each domain. The overall score for research importance was then measured as the sum of these scores divided by the number of domains. The association of these scores with values for priorities, responder’s identification as patients, osteopathic practitioners, educators, researchers or policymakers or country settings was computed using regression analysis. The dependent variable was the score for importance. The independent/explanatory variables were dichotomised representative groups (ie, patients, practitioners (ref.), students, educators, researchers, policy makers), dichotomised priority groups (ie, conservative (ref.), sceptic, enthusiast) and dichotomised countries (df=17, UK=ref.). The domain score corresponds to the constant of the model. Significant level for associations of independent variables was set for coefficients being significantly different to null with p<0.01 without adjustment for multiple testing. No prior sample size calculation was made as the sampling method aimed to include as many participants as possible to enable subgroup analysis.

The protocol was registered prior to data collection and is made publicly available^26^ (https://zenodo.org/records/8322740). All analyses were conducted using STATA (StataCorp, 2017. Stata Statistical Software: Release 15. StataCorp LLC, College Station, Texas, USA). Full data, coding for analysis and statistical outputs are made available on Zenodo^51^ (https://zenodo.org/doi/10.5281/zenodo.14826001).

### Patient and public involvement

Patients and public were not involved in designing the study. The public (ie, UK and Switzerland) was first solicited when testing the survey questionnaire using a think aloud approach that also evaluated the burden and time required to participate. They then contributed to recruiting patient representative responders, which made it possible to analyse and take their perspectives into account when setting research priorities.

## Results

### Responses and rate of completion

This large-scale consultation captured perspectives from 2229 participants across 42 countries, representing the most comprehensive investigation of osteopathic research priorities to date.

The surveys were completed in French (n=773; 34.7%), German (n=497; 22.3%), English (n=355; 15.9%), Spanish (n=200; 9.0%), Italian (n=190; 8.5%), Portuguese (n=105; 4.7%), Dutch (n=48; 2.1%), Finnish (n=46; 2.1%) and Swedish (n=15; 0.7%). The median time to complete the survey was 18.3 min (range 3.3 min to 10 days).

There was a total of 4050 clicks to begin the survey. 47 clicks were from responders who declined to participate further; 18 (0.4%) for already having completed the survey, and 29 (0.7%) who reported preferring not to participate. Given the survey was anonymous, there is no way of knowing how many people returned and started the survey multiple times before ending it. Only surveys that were completed to the end were included (n=2229). The proportion of surveys that were opened without being completed after 10 days was constant over the recruitment period. Overall, answers from incomplete surveys revealed lower scores of importance for principal research domains (range from −0.18 to −0.10) and for nine subdomains (range from −0.16 to −0.08). A large majority of responders (N=1830; 82.1%) were able to provide their evaluation of importance for all 35 domains and subdomains.

### Description of studied population

Responders identified themselves as having their main experience with osteopathic care in a total of 42 countries (online supplemental material A; table A). Responders identified themselves mainly from Europe (n=2023; 90.8%). Among the 189 (8.4%) responders from other countries, 79 (3.5%) were from Brazil and 68 (3.1%) from Canada; two countries whose professional associations are associate members of OE.

Most responders identified themselves primarily as practitioners (42.1%), then as students (17.4%), then educators (13.5%), then patients (7.4%), then researchers (5.0%) and finally as policy makers (4.3%). The UK had the highest proportion of patient representatives (25.8%), Switzerland had the highest proportion of practitioners (66.3%), Finland had the highest proportion of students (37.0%), Brazil had the highest proportion of educators (21.5%) and Canada had the highest proportion of researchers (11.8%) and policy makers (10.3%).

The responders were representative of the overall population’s gender with over half (51.4%) of responders identifying themselves as cisgender women and 1.1% as non-cisgender. Apart from Belgium, Spain, Italy and France, all other countries had a majority of women responders. The age group that was best represented was 35–49 years (38%). The French responders were younger than other responders (62.2%<35 years) and responders from the UK were older (17.7%≥65 years). The 10 countries which had more than 50 responders were: Belgium, Brazil, Canada, Switzerland, Germany, Spain, Finland, France, Italy and the UK. When using the Osteopathic International Alliance (OIA) reported number of osteopaths in these countries,^3^ response rates per 1000 osteopaths varied considerably between countries (range 15–568).

### Objective 1: homogeneity/heterogeneity of importance accorded to values when assessing research importance between interested parties

**Figure 2:**
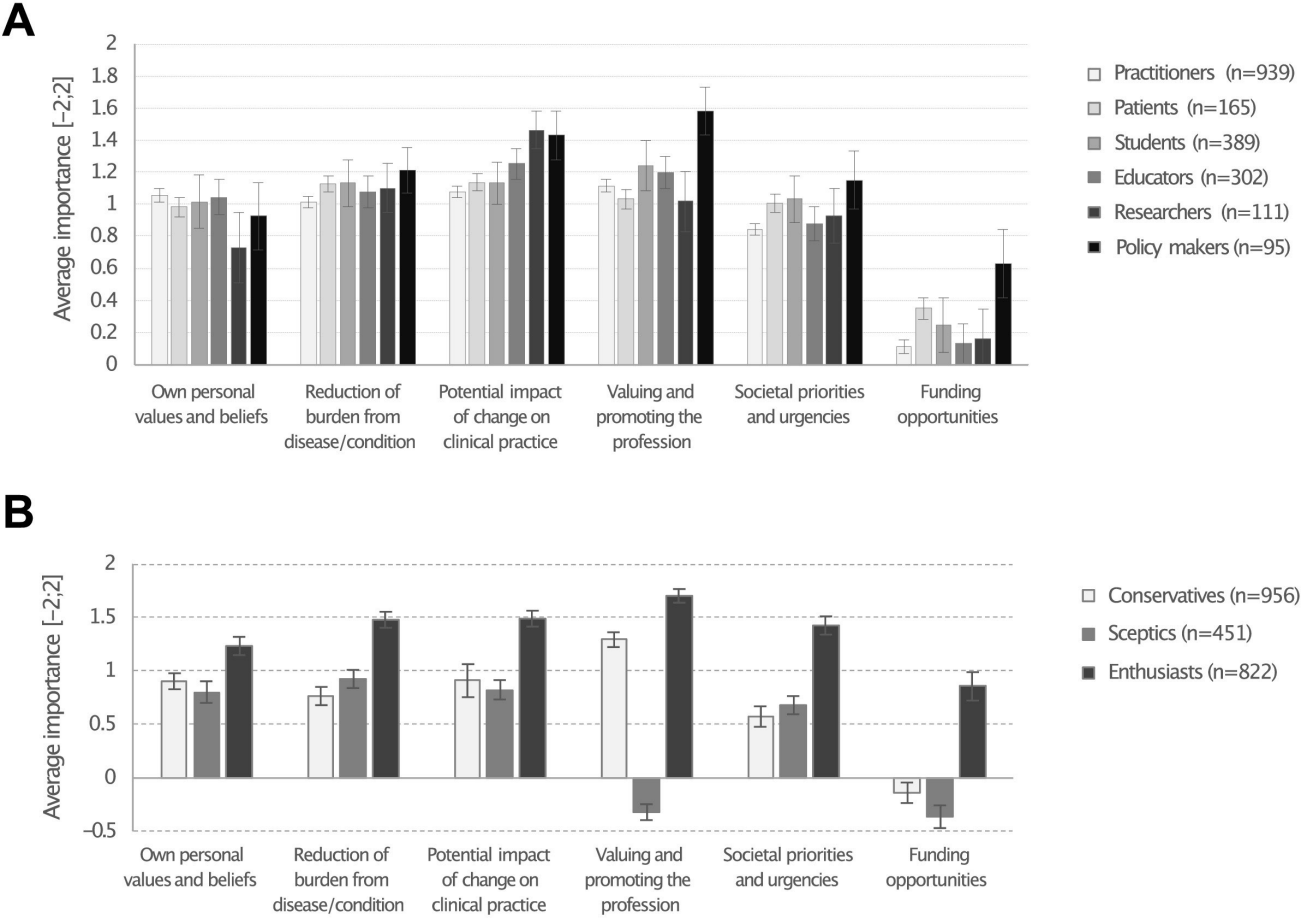
(A) Average scores of importance accorded to criteria for setting priorities; (B) Rresponders’ profiles for setting priorities. Error bars correspond to 95% CI.

Apart from *funding opportunities*, responders scored average similar values of importance for all other criteria for setting priorities (figure 2A). The maximum score being 2.0 points, researchers (1.4 points) accorded more importance to *Potential impact of change on clinical practice* than patients (1.14 points), practitioners (1.09 points) and students (1.13 points), and less importance to *Personal values and beliefs* compared with practitioners (1.05 vs 0.73). Policy makers accorded more value than any other expert group to *Valuing and promoting the profession* (1.58 points vs 1.02–1.23) and to *Funding opportunities* (0.63 points vs 0.12–0.37). Patients (1.01), students (1.03) and policy makers (1.15) accorded more importance to *Societal priorities and urgencies* than practitioners (0.84).

Three distinct approaches to priority-setting emerged from the latent class analysis: conservative (42.9%), sceptic (20.2%) and enthusiast (36.9%), reflecting fundamental differences in how interested parties conceptualise research value (figure 2B). The first group prioritised the importance of valuing and promoting the profession over societal priorities and urgencies and funding opportunities (*Conservatives*). The second group corresponded to those who did not accord importance to valuing and promoting the profession (*Sceptics*). The third group accorded more importance to all values including for societal priorities and funding opportunities (*Enthusiasts*). Table 1 describes characteristics for each group.

**Table 1:** Description of responders depending on their profile for setting priorities in research; n (%)

	**Conservative (**n=956)	**Sceptic (**n=451)	**Enthusiast (**n=822)
Gender			
Cisgender—women	487 (50.9)	200 (44.3)	459 (55.8)
Cisgender—men	440 (46.0)	224 (49.7)	329 (40.0)
Transgender, bigender, agender	7 (0.7)	8 (1.8)	10 (1.2)
Age (years)			
<35	309 (32.3)	151 (34.5)	275 (33.4)
35–49	369 (38.6)	171 (37.9)	307 (37.3)
50–64	224 (23.4)	91 (20.2)	194 (23.6)
≥65	40 (4.2)	29 (6.4)	37 (4.5)
Interested parties			
Patient	57 (6.0)	40 (8.9)	68 (8.3)
Practitioner	435 (45.5)	198 (43.9)	306 (37.2)
Student	158 (16.5)	68 (15.1)	163 (19.8)
Educator	135 (14.1)	52 (11.5)	115 (14.0)
Researcher	46 (4.8)	24 (5.3)	41 (5.0)
Policy maker	37 (3.9)	10 (2.2)	48 (5.8)
Preferred not to answer	88 (9.2)	59 (13.1)	81 (9.8)

### Objective 2: internal validity of the PROCare Eye

The entire construct for domains and subdomains for the PROCare Eye (figure 1) showed a high internal validity with an overall Cronbach’s α of 0.911 and a KMO measure of sampling adequacy of 0.918. PCA identified seven factors from all seven domains and 28 subdomains put together (table 2). One factor grouped all the domains together and the six others identified questions from each separate domain except for ‘process of care’ and ‘healthcare management’, which were identified as a single factor and were grouped together. All subdomains were grouped correctly together in their own domain except for ‘research culture’ that responders identified as belonging more to ‘methodology research’ rather than to ‘education research’.

**Table 2:** Internal validity of construct for research priorities (N=1830)

	KMO	Uniqueness	Eigenvalue	CFA rank*
Process of care† (D1)	0.922	0.539	8.791†	I
Effectiveness and efficacy (D1.1)	0.916	0.676		4
Patient safety (D1.2)	0.941	0.698		1
Patient management (D1.3)	0.926	0.444		22
Decision making (D1.4)	0.946	0.615		12
Psychology (D1.5)	0.931	0.666		9
Healthcare management† (D2)	0.934	0.498	8.791†	VI
Service user perspective (D2.1)	0.949	0.567		15
Professional development (D2.2)	0.947	0.554		14
Cost-effectiveness (D2.3)	0.943	0.616		21
Service organisation (D2.4)	0.961	0.625		17
Quality improvement (D2.5)	0.956	0.599		4
Digital health (D2.6)	0.955	0.623		28
Population health (D3)	0.925	0.447	1.184	IV
Physical activities and mobility (D3.1)	0.889	0.380		2
Public awareness and education (D3.2)	0.919	0.401		3
Epidemiology (D3.3)	0.917	0.494		19
Distinct populations (D3.4)	0.925	0.453		26
Education research (D4)	0.953	0.525	1.876	III
Research culture (D4.1)	0.944	0.466		20
Knowledge transfer (D4.2)	0.934	0.440		4
Undergraduate education (D4.3)	0.918	0.540		10
Continuing education (D4.4)	0.932	0.445		8
Basic sciences (D5)	0.893	0.419	1.876	I
Theoretical concepts (D5.1)	0.917	0.562		24
Biomechanics, anatomy, physiology (D5.2)	0.881	0.406		4
Osteopathic manual treatment mechanisms (D5.3)	0.840	0.380		10
Osteopathic diagnosis (D5.4)	0.871	0.474		12
Methodology in research (D6)	0.919	0.385	2.307	V
Methodology improvement (D6.1)	0.876	0.306		22
Outcome measures (D6.2)	0.858	0.346		18
Participatory research (D6.3)	0.944	0.568		16
Social justice research (D7)	0.927	0.268	1.476	VII
Engagement and advocacy (D7.1)	0.858	0.171		27
Inclusive practice and education (D7.2)	0.868	0.195		25

* CFA rank is defined by the ‘importance score’ or the constant value from the Model for Research importance in online supplemental file B. Roman numbers correspond to domains, Arabic numbers correspond to subdomains.

† Subdomains from process of care and healthcare management were identified as coming from a single factor.

CFA, confirmatory factorial analysis; KMO, Kaiser-Meyer-Olkin measure of sampling adequacy.

Confirmatory factorial analysis (online supplemental material B; figure B) revealed that measures lacked consistency in correctly modelling the construct for ‘research importance’. The model centred around the construct of ‘research importance’ was able to explain 76.5% of the observed variance (R^2^), with a root mean squared error of approximation of 0.071 suggesting acceptable but poor fit. Both Comparative Fit Index (0.742) and Tucker-Lewis Index (0.723) were below the threshold of 0.9. Sensitivity analysis adding the identity as ‘conservatives’, ‘sceptics’ or ‘enthusiasts’ from the latent class analysis to the model did not help reach the set threshold.

### Objective 3: research priorities

#### Principle research domains

Over half of the responders found *process of care*, *population health*, *education research* and *basic sciences* to be very important, and *healthcare management*, *methodology in research* and *social justice research* to be either important or very important.

Ranks of importance for principal research domains are provided in table 3 based on the crude average scores of importance. This order of priority remains unchanged when using ‘importance scores’ from the CFA (table 2) that accounted for answers from all 2229 responders and provides weighted scores based on the overall concept of ‘research importance’; the only difference being *basic science* also taking the first place with *process of care*.

**Table 3:** Scores for priorities in research domains and subdomains (N=2229)

	n*	Median score	Crude score, average (SD)	Rank no
Process of care (D1)	2207	2	1.575 (0.776)	I
Effectiveness and efficacy (D1.1)	2213	2	1.462 (0.704)	10
Patient safety (D1.2)	2220	2	1.716 (0.591)	1
Patient management (D1.3)	2169	1	1.111 (0.829)	21
Decision making (D1.4)	2183	2	1.385 (0.730)	13
Psychology (D1.5)	2200	2	1.394 (0.714)	12
Healthcare management (D2)	2188	1	0.946 (1.000)	VI
Service user perspective (D2.1)	2114	1	1.185 (0.720)	16
Professional development (D2.2)	2183	1	1.284 (0.772)	14
Cost-effectiveness (D2.3)	2182	1	0.904 (0.858)	26
Service organisation (D2.4)	2189	1	1.138 (0.849)	18
Quality improvement (D2.5)	2194	2	1.469 (0.686)	9
Digital health (D2.6)	2178	0	0.217 (1.035)	28
Population health (D3)	2217	2	1.348 (0.832)	IV
Physical activities and mobility (D3.1)	2223	2	1.641 (0.626)	2
Public awareness and education (D3.2)	2223	2	1.539 (0.664)	6
Epidemiology (D3.3)	2210	1	1.123 (0.818)	20
Distinct populations (D3.4)	2186	1	0.895 (0.877)	27
Education research (D4)	2223	2	1.396 (0.777)	III
Research culture (D4.1)	2170	1	1.078 (0.841)	22
Knowledge transfer (D4.2)	2201	2	1.497 (0.650)	8
Undergraduate education (D4.3)	2178	2	1.433 (0.743)	11
Continuing education (D4.4)	2201	2	1.510 (0.690)	7
Basic sciences (D5)	2224	2	1.519 (0.744)	II
Theoretical concepts (D5.1)	2198	1	0.951 (0.886)	24
Biomechanics, anatomy, physiology (D5.2)	2220	2	1.627 (0.660)	3
Osteopathic manual treatment mechanisms (D5.3)	2218	2	1.572 (0.727)	4
Osteopathic diagnosis (D5.4)	2214	2	1.541 (0.762)	5
Methodology in research (D6)	2212	1	1.162 (0.884)	V
Methodology improvement (D6.1)	2157	1	1.141 (0.833)	17
Outcome measures (D6.2)	2163	1	1.137 (0.787)	19
Participatory research (D6.3)	2171	1	1.231 (0.803)	15
Social Justice Research (D7)	2201	1	0.889 (1.027)	VII
Engagement and advocacy (D7.1)	2160	1	0.920 (1.002)	25
Inclusive practice and education (D7.2)	2159	1	1.058 (0.986)	23

Scores ranged from ‘very unimportant’ to ‘very important’ [−2; 2].

* n=number of responders after excluding those who preferred not to answer the question.

**Figure 3:**
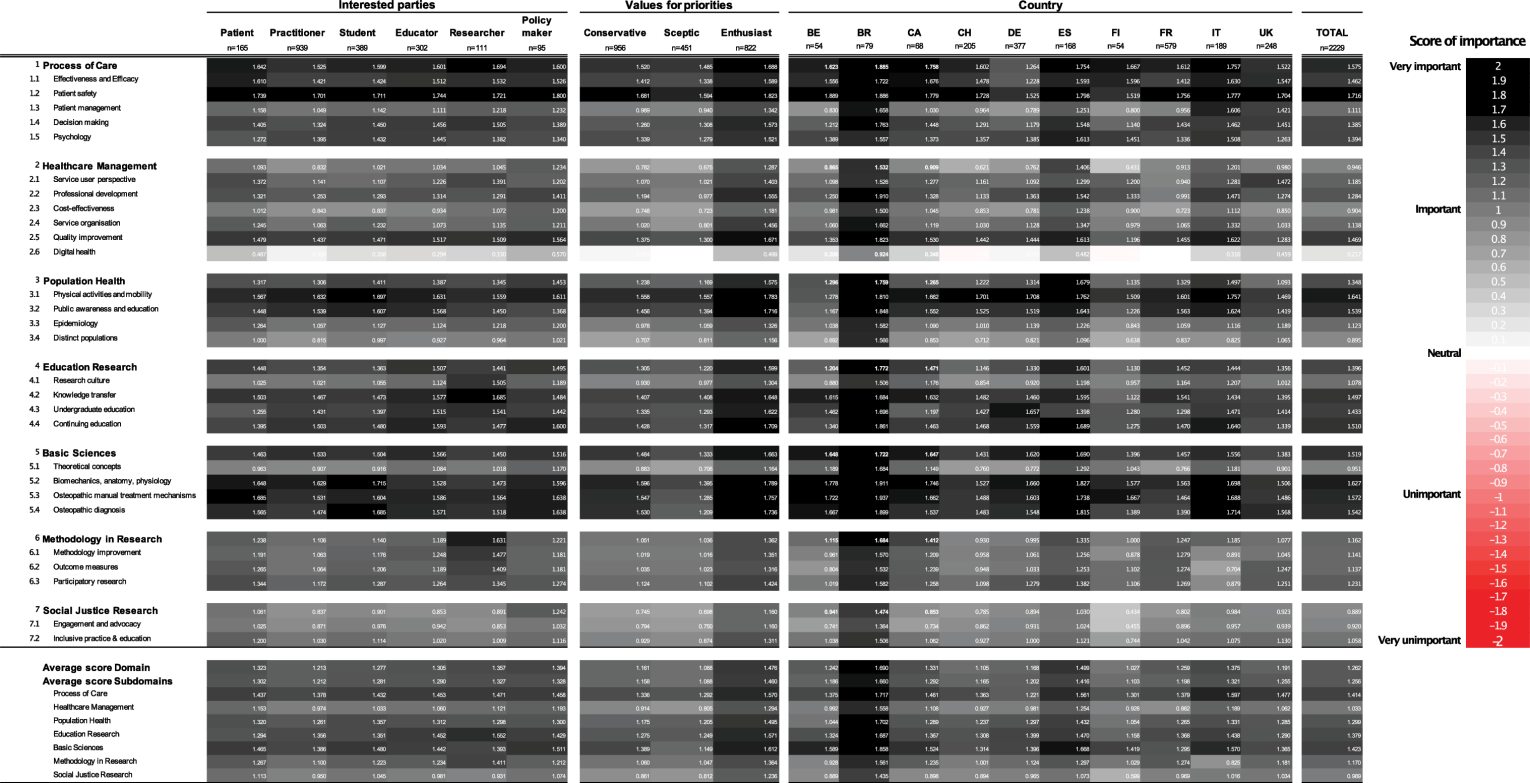
Principal research domains and subdomains priorities. BE, Belgium; BR, Brazil; CA, Canada; CH, Switzerland; DE, Germany; ES, Spain; FI, Finland; FR, France; IT, Italy.

Discrepancies in opinions concerning priorities were highest for *Social justice research* (SD=1.027) and lowest for *Basic sciences* (SD=0.744). Details for measured average scores are provided in the last column of figure 3.

#### Research subdomains

Of 28 subdomains, 13 were judged as ‘very important’ by more than half of the responders, 14 as at least ‘important’, and 1 as at least ‘neutral’ (table 3). Ranking methods (ie, mean crude score—table 3, CFA—table 2) identified *patient safety* and *physical activities and mobility* as the two most important research subdomains, and *digital health* as the least important. CFA accorded more importance to *public awareness and education*, *quality improvement*, *effectiveness and efficacy* and *knowledge transfer* than crude means; thereby revealing the versatility of subdomain ranking depending on the statistical method that was used.

Discrepancies in opinions about importance were the highest for *digital health* (SD=1.035), *engagement and advocacy* (SD=1.002), *inclusive practice and education* (SD=0.986), *theoretical concepts* (SD=0.886) and *cost-effectiveness* (SD=0.858). Details for each subdomain are provided in the last column of figure 3.

#### Topics

Participants were given the opportunity to make additional suggestions for research topics within each *domain*. 240 open text contributions were entered. After redundant or irrelevant (ie, defined as not possibly being a research topic) items were removed, 26 topics were identified. All of these could potentially be placed on the PROCare Eye (figure 1).

Content analysis identified four topics that could fit into *healthcare management*: ‘professionalism’ (*professional development—policy development*), ‘health ethics and integrity’ (*professional development—policy development*), ‘return to work after being off the register’ (*service organisation—workforce*), ‘preconsultation’ (*service organisation—scope of practice*). Three topics could fit into *population health*: ‘built environment favouring a mobile lifestyle’ (*physical activities and mobility—sedentary lifestyle prevention*), ‘favour outdoor activities’ (*physical activities and mobility—sedentary lifestyle prevention*) and ‘salutogenesis’ (*epidemiology—causes and evolution of conditions*). Two topics could fit into *process of care*: ‘emotional intelligence’ (*decision making—case explanation and subjectivity*) and ‘cognitive biases’ (*decision making—case explanation and subjectivity*). Two topics could fit into *education research*: ‘practitioner supervision’ (*continuing education—clinical and practical demands*), ‘practitioner self-reflection on process’ (*continuing education—clinical and practical demands*). 10 suggestions were disciplines rather than research topics: ‘taxonomy’, ‘anthropology’, ‘phenomenology’, ‘philosophy’, ‘psychopathology and pathophysiology’, ‘marketing’, ‘microbiology/microbiome’, ‘nutrition’, ‘naturopathy’, ‘bioenergetics/bioresonance’. Two suggestions were project development rather than research: ‘creation of an oversight body (ethics committee, order, general counsel)’ and ‘humanitarian aid’. Lastly, three topics focused on health conditions: ‘Explore complaints’, ‘natural history of common MSK conditions’ and ‘trauma clinical investigation, care and research’.

Table 4 gives the list of the top topic selections from each of the principal research domains. Three topics were selected by half or more of responders: *hands on techniques* (54.7%), *sedentary lifestyle prevention* (51.0%) and *neurophysiological effects of osteopathic manual treatments* (50.0%).

**Table 4:** Top choices for research topics within each principal research domain

Domain	Rank	Research subdomain	Topic	Score
Process of care	1	Effectiveness and efficacy	Hands on techniques	54.7%
	2	Patient management	Therapeutic alliance and communication skills	34.7%
	3	Decision making	Clinical diagnosis	34.7%
	4	Patient management	Patient-centred care	31.0%
	5	Effectiveness and efficacy	Psychosocial aspect of care	22.3%
Healthcare management	1	Professional development	Professional identity	34.8%
	2	Service user perspective	Patient preferences and prioritisation	30.6%
	3	Service user perspective	Experience and perception of care	27.6%
	4	Cost-effectiveness	Cost–benefit analysis of services	25.8%
	5	Service organisation	Access to care	25.5%
	5	Service organisation	Care coordination and multi-disciplinary dynamics	25.5%
Population health	1	Physical activities and mobility	Sedentary lifestyle prevention	51.0%
	2	Public awareness and education	Health promotion	37.5%
	3	Physical activities and mobility	Behavioural change in physical activities	35.4%
	4	Public awareness and education	Public accessibility of evidence-based information	23.6%
Education research	1	Continuing education	Quality of training and education	39.7%
	2	Knowledge transfer	Critical thinking	38.6%
	3	Knowledge transfer	Best practice integration into clinical routine	37.8%
	4	Research culture	Evidence-based education	27.6%
Basic sciences	1	Osteopathic manual treatment mechanisms	Neurophysiological effects of OMT	50.0%
	2	Osteopathic manual treatment mechanisms	Neuroendocrine and immunological responses to OMT	35.8%
	3	Biomechanics, anatomy, physiology	Pain/touch physiology	32.2%
	4	Theoretical concepts	Principle updates in line with evidence	29.1%
Methodology research	1	Methodology improvement	Innovative methods and research framework for NPI	37.4%
	2	Outcome measures	Patient centredness of investigation methods	28.0%
	3	Outcome measures	Clinical meaningfulness of investigation methods	26.2%
Social justice research	1	Engagement and advocacy	Public health priorities	31.5%
	2	Inclusive practice and education	Equity	29.3%

NPI, non-pharmaceutical interventions; OMT, osteopathic manual treatment.

**Figure 4:**
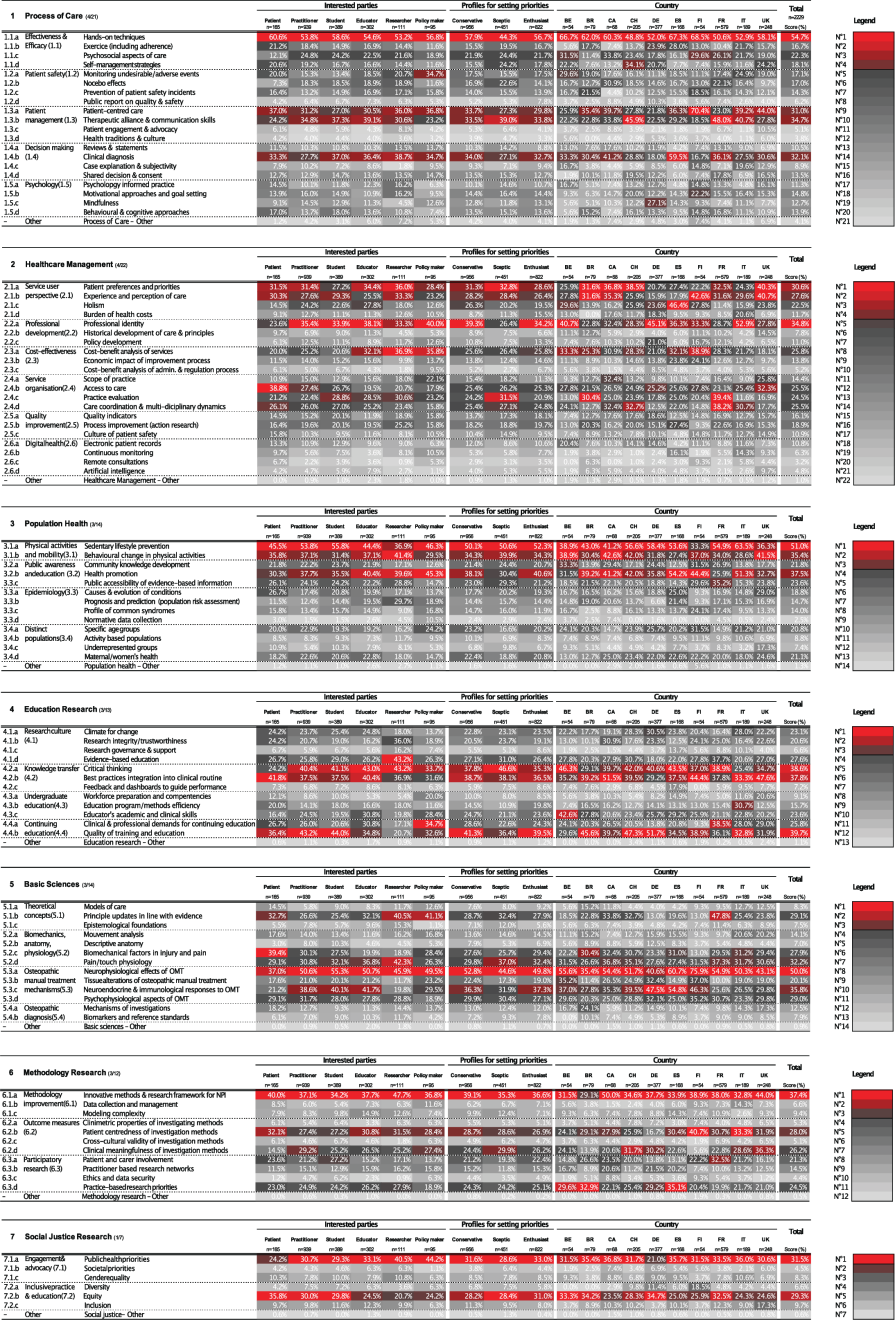
Topic priorities by principal research domains (N=2229). BE, Belgium; BR, Brazil; CA, Canada; CH, Switzerland; DE, Germany; ES, Spain; FI, Finland; FR, France; IT, Italy; NPI, non-pharmaceutical intervention; OMT, osteopathic manual treatment.

At the other end of the spectrum (figure 4, last column), the three least chosen topics were *normative data collection* (epidemiology; 2.5%), *remote consultations* (digital health; 3.2%) and *health traditions and culture* (patient management; 3.8%). Responders also expressed little interest in developing further research in *descriptive anatomy* (7.0%), *epistemological foundations* (7.5%) and *historical development of care and principles* (7.8%), *societal priorities* (4.5%), *artificial intelligence* (4.8%) or *models of care* (8.3%).

### Objective 4: contextual factors’ association to research prioritisation

Subgroup descriptive results for principal research domains and subdomains are provided in figure 3, and for research topics by principal research domains in figure 4.

Compared with the UK, conservative practitioners, ‘*research importance*’ was scored higher by enthusiasts (+0.3 points (95% CI 0.26 to 0.34; p<0.001)) and by responders from Brazil (+0.29 points (95% CI 0.19 to 0.39; p<0.001)), and scored lower by sceptics (−0.07 points (95% CI −0.12 to −0.03; p=0.001)).

Enthusiast responders scored higher in all principal research domains; *process of care* +0.23 points (95% CI 0.19 to 0.27; p<0.001), *healthcare management* +0.36 points (95% CI 0.31 to 0.41; p<0.001), *population health* (+0.30 points (95% CI 0.25 to 0.35; p<0.001)), *educational research* (+0.29 points (95% CI 0.24 to 0.34; p<0.001)), *basic sciences* (+0.20 points (95% CI 0.14 to 0.25; p<0.001)), *methodology in research* (+0.33 points (95% CI 0.27 to 0.39; p<0.001)) and *social justice research* (+0.41 points (95% CI 0.32 to 0.50; p<0.001)). Sceptics scored lower in *healthcare management* (−0.13 points (95% CI −0.19 to −0.08; p<0.001)) and *basic sciences* (−0.22 points (95% CI −0.27 to −0.16; p<0.001)).

Brazilian responders accorded more importance to research in *process of care* (+0.15 points (95% CI 0.04 to 0.26; p=0.009)), *healthcare management* (+0.36 points (95% CI 0.23 to 0.48; p<0.001)), *population health* (+0.31 points (95% CI 0.18 to 0.45; p<0.001)), *education research* (+0.28 points (95% CI 0.15 to 0.41; p<0.001)), *basic sciences* (+0.39 points (95% CI 0.25 to 0.52; p<0.001)) and *methodology in research* (+0.27 points (95% CI 0.11 to 0.43; p=0.001)). Belgian responders accorded more importance to research in *basic sciences* (+0.24 points (95% CI 0.09 to 0.40; p=0.002)) but less importance to research in *population health* (−0.20 points (95% CI−0.36 to −0.05; p=0.009)). French responders accorded less importance to research in *process of care* (−0.09 points (95% CI −0.16 to −0.03; p=0.006)) and *healthcare management* (−0.16 points (95% CI −0.28 to −0.03; p<0.001)). German responders accorded less importance to research in *process of care* (−0.27 points (95% CI −0.34 to −0.20; p<0.001)). Italian responders accorded more importance to research in *basic sciences* (0.16 points (95% CI 0.06 to 0.26; p=0.002)), but less importance to *methodology in research* (−0.39 points (95% CI −0.51 to −0.26; p<0.001)). Spanish responders accorded more importance to research in *basic sciences* (0.26 points (95% CI 0.15 to 0.36; p<0.001)).

Finally, researchers accorded more importance in research in *education research* (+0.17 points (95% CI 0.08 to 0.27; p<0.001)) and in *methodology in research* (+0.23 points (95% CI 0.11 to 0.35; p<0.001)).

## Discussion

### Summary of findings

The present study yields four principal findings that advance understanding of research priorities in osteopathic care. First, interested parties conceptualise research importance through distinctly different lenses—conservative, sceptic and enthusiast approaches—which transcend traditional professional boundaries. Second, despite these different philosophical approaches, strong consensus emerged around core priorities including patient safety, physical activity promotion and understanding treatment mechanisms. Third, the validated PROCare framework provides a novel taxonomic structure for systematically evaluating research priorities across diverse interest groups. Fourth, while geographic variations exist in priority emphasis, fundamental agreement on key research domains suggests potential for internationally coordinated research strategies.

The identification of three distinct interested parties profiles—conservatives (42.9%), sceptics (20.2%) and enthusiasts (36.9%)—demonstrates fundamental differences in how research value is conceptualised across the profession. This aligns with Fleurence and Torgerson’s^24^ work highlighting how underlying values shape research prioritisation, though our findings suggest these groupings transcend traditional professional boundaries (ie, distinction between patients, practitioners, educators, researchers and policy makers). Despite these differing philosophical approaches, strong consensus emerged around core priorities. The strong emphasis on patient safety and treatment mechanisms aligns with priorities identified in other musculoskeletal care settings, as demonstrated by Andersen *et al* in their recent analysis of chronic musculoskeletal pain research priorities across healthcare sectors.^52^ However, the notably low prioritisation of digital health innovation (ranked 28th of 28 subdomains) contrasts with broader healthcare research agendas and may reflect a tension between profession-specific concerns and wider healthcare system priorities. The geographical variations in priority emphasis are likely to reflect differences in professional regulation and healthcare system integration. For example, Brazilian responders accorded significantly higher importance to most research domains compared with other regions (+0.15 to +0.39 points higher than UK baseline, p<0.001). Nevertheless, the fundamental agreement on core priorities, particularly around process of care and basic sciences, suggests potential for coordinated international research strategies despite these contextual differences. While our findings demonstrate clear patterns in research priorities, the forecasted 115% increase in other musculoskeletal disorder cases between 2020 and 2050 underscores the urgency of addressing these research needs systematically.^53^ This projected increase, driven primarily by population growth and ageing, highlights the importance of developing evidence-based approaches to manage growing demand for osteopathic services.

Our findings should be interpreted within the broader context of health priority-setting approaches. A recent analysis of national health priorities across 145 countries found little correlation between priorities and disease burden as measured by disability-adjusted life years (DALYs).^54^ Similar to our identification of distinct *conservative*, *sceptic* and *enthusiast* approaches to research prioritisation, they found that priority-setting was influenced by multiple factors beyond pure burden metrics. This suggests that discrepancies between measured burden and research priorities may be a systemic feature of health priority-setting rather than unique to osteopathic care. The systematic deprioritisation of musculoskeletal conditions that predominantly contribute to years lived with disability rather than mortality has been observed in national priority-setting. Oliveira *et al*^54^ found that low back pain and headache disorders had among the lowest proportions of nomination as health priorities (6%) despite their high disease burden. Osteopaths might find it more important to understand mechanisms and process of care than catering to population health, possibly because that’s how they were initially trained to think. This underlines the importance of favouring the integration of research culture at all levels within allied health professions.^2^

Our findings reveal critical insights into interested parties’ engagement within osteopathic research prioritisation. The identification of distinct value-based profiles suggests the need for more nuanced approaches to research engagement and implementation. Conservative interested parties demonstrate strong alignment with profession-strengthening research priorities, while sceptics emphasise methodological rigour and evidence-based approaches. Enthusiasts, in contrast, show openness to integrating multiple research perspectives, potentially serving as bridges between different interested parties. Importantly, we found substantial alignment in research priorities across different interested parties, creating valuable opportunities for cross-professional collaboration. This alignment could facilitate joint research initiatives between clinicians and academics, foster patient–practitioner collaborative projects and support the development of multi-centre international studies. Such collaborative approaches could significantly enhance the quality and relevance of osteopathic research while promoting interdisciplinary knowledge exchange. The strong consensus on core priorities provides a clear pathway for implementing research strategies across different timeframes. In the short term, focusing on patient safety and treatment mechanisms addresses immediate professional needs. Medium-term strategies can emphasise the development of physical activity interventions, while long-term planning can incorporate innovative methodologies as the evidence base expands. This temporal approach allows for systematic development of research programmes while maintaining interested parties’ engagement. These findings underscore the importance of a balanced approach to research implementation that acknowledges diverse interested parties’ perspectives while maintaining focus on agreed priorities. Such an approach could help ensure that osteopathic research remains both rigorous and relevant to clinical practice while fostering continued engagement across the profession’s diverse interested parties.

This study aligns with recent work in pain research prioritisation, which has emphasised the need for systematic interested parties’ engagement in setting research agendas. The European Pain Federation’s (EFIC) comprehensive priority-setting exercise^55^ reveals several key parallels with our findings, particularly around the emphasis on understanding fundamental mechanisms and taking an integrated biopsychosocial approach. The geographic variations in research priorities identified in our study find interesting parallels with those found by the EFIC group, where they noted ‘regional differences in European countries with respect to medical, social and political emergencies, including access to resources, potential for clinical/basic research and autonomy of practice’. This suggests that research priority-setting must carefully balance local contexts with the need for coordinated international strategies.

### Strengths and limitations of the study

This study has several notable strengths. Foremost, with 2229 participants, it represents the largest international survey of osteopathic research priorities conducted to date, providing robust statistical power for comprehensive subgroup analyses. Our novel sequential exploratory approach, which combined qualitative framework development with quantitative validation, enhanced methodological rigour and deepened our understanding of priority-setting processes. The inclusion of diverse interested parties—spanning patients, practitioners, educators, researchers and policymakers—enabled a comprehensive perspective that captures the breadth of views within the osteopathic community. Furthermore, our validated statistical approach to identifying priority-setting profiles advanced theoretical understanding of how different interested parties conceptualise research value. The availability of the survey in nine languages facilitated broad geographic representation, strengthening the international relevance of our findings.

However, several limitations warrant consideration. First, while the umbrella review offers valuable insights, having conducted it by a single researcher introduces significant risks of bias and limitations. This was, however, mitigated by having 18 experienced researchers with diverse expertise fields in research review the entire process thoroughly. Nevertheless, subjectivity and confirmation bias cannot be entirely ruled out, and the theoretical framework should not be considered as entirely theory driven. Second, our reliance on convenience sampling through professional networks may have introduced selection bias towards research-engaged participants, potentially overlooking views of those less connected to research activities. Third, the cross-sectional design, while efficient for capturing current perspectives, prevents causal inference regarding how research priorities form and evolve over time. Fourth, response rates varied considerably between countries (ranging from 15 to 568 per 1000 osteopaths), which may limit generalisability in regions with lower representation. Some countries (ie, the USA, Australia) or interested parties (ie, patients) are under-represented. Patient perspectives were predominantly drawn from the UK (38.8% of patient responses), potentially failing to capture the full spectrum of international patient views. Additionally, our reliance on self-reported ‘interested party’ categorisation may not perfectly align with formal roles, as individuals often occupy multiple positions within the profession. Finally, the identified priorities are those perceived by various interested parties. Such priorities may not necessarily lead to the most impactful research.

### Practical implications

These findings have immediate implications for research strategy and funding allocation:

1. Research funding: Funding bodies should consider adopting the validated PROCare framework to evaluate proposal alignment with interested parties’ priorities, particularly emphasising: (a) patient safety investigations; (b) physical activity promotion interventions and (c) treatment mechanism studies.
2. Research strategy: The identification of distinct priority-setting profiles suggests need for: balanced representation of conservative, sceptic and enthusiast perspectives on funding panels; explicit consideration of how proposed research addresses different interested parties’ values; and development of metrics capturing multiple concepts of research value. It also suggests that funders could gain in putting forward priority-specific project proposals.
3. International coordination: The strong cross-national consensus on core priorities provides a foundation for: (a) development of international research networks focused on high-priority domains; (b) standardised outcome measures enabling cross-national comparison and (c) coordinated funding strategies maximising resource utilisation.
4. Professional development—educational institutions should incorporate: (a) training in research priority assessment using the PROCare framework; (b) understanding of different value-based approaches to priority setting; and (c) skills in interested parties engagement and priority consensus building.

## Conclusions

This study provides a proposition of a framework for research priorities within osteopathic care. The PROCare framework offers a robust structure for advancing evidence-based practice while respecting diverse interested parties’ perspectives. It can be used for strategic research planning at national and international levels, helping assure research addresses the full scope of the profession.

## Supplementary material

10.1136/bmjopen-2025-100757 online supplemental file 1

10.1136/bmjopen-2025-100757 online supplemental file 2

## Data Availability

Data are available in a public, open access repository.

## References

[1] Goorts K, Dizon J, Milanese S (2021). The effectiveness of implementation strategies for promoting evidence informed interventions in allied healthcare: a systematic review. BMC Health Serv Res.

[2] Slade SC, Philip K, Morris ME (2018). Frameworks for embedding a research culture in allied health practice: a rapid review. Health Res Policy Syst.

[3] Carnes D, Ellwood J, Hunt C (2020). The oia global report: global review of osteopathic medicine and osteopathy 2020.

[4] CEN (2015). Osteopathic Healthcare Provision EN 16686:2015.

[5] Osteopathy Europe (2023). Regulation of the osteopathic profession in europe – an overview.

[6] Wong EC, Maher AR, Motala A (2022). Methods for Identifying Health Research Gaps, Needs, and Priorities: A Scoping Review. Rand Health Q.

[7] Duffett L (2017). Patient engagement: What partnering with patient in research is all about. Thromb Res.

[8] Brett J, Staniszewska S, Mockford C (2014). Mapping the impact of patient and public involvement on health and social care research: a systematic review. Health Expect.

[9] World Health Organization (2016). Patient Engagement.

[10] Graffigna G (2021). Patient engagement as a crucial asset of preclinical biomedical research. EBioMedicine.

[11] Pratte M-M, Audette-Chapdelaine S, Auger A-M (2023). Researchers’ experiences with patient engagement in health research: a scoping review and thematic synthesis. Res Involv Engagem.

[12] Elberse JE, Caron-Flinterman JF, Broerse JEW (2011). Patient-expert partnerships in research: how to stimulate inclusion of patient perspectives: Stimulate inclusion of patient perspectives. Health Expect.

[13] Grill C (2021). Involving stakeholders in research priority setting: a scoping review. Res Involv Engagem.

[14] Terry RF, Charles E, Purdy B (2018). An analysis of research priority-setting at the World Health Organization – how mapping to a standard template allows for comparison between research priority-setting approaches. Health Res Policy Sys.

[15] Wilson P, Mathie E, Keenan J (2015). ReseArch with Patient and Public invOlvement: a RealisT evaluation – the RAPPORT study. Health Serv Deliv Res.

[16] Manafò E, Petermann L, Vandall-Walker V (2018). Patient and public engagement in priority setting: A systematic rapid review of the literature. PLoS ONE.

[17] Rushton AB, Fawkes CA, Carnes D (2014). A modified Delphi consensus study to identify UK osteopathic profession research priorities. Man Ther.

[18] Mota da Silva T, da Cunha Menezes Costa L, Garcia AN (2015). What do physical therapists think about evidence-based practice? A systematic review. Man Ther.

[19] Weber V, Rajendran D (2018). UK trained osteopaths’ relationship to evidence based practice - An analysis of influencing factors. Int J Osteopath Med.

[20] Sundberg T, Leach MJ, Thomson OP (2018). Attitudes, skills and use of evidence-based practice among UK osteopaths: a national cross-sectional survey. BMC Musculoskelet Disord.

[21] Leach MJ, Sundberg T, Fryer G (2019). An investigation of Australian osteopaths’ attitudes, skills and utilisation of evidence-based practice: a national cross-sectional survey. BMC Health Serv Res.

[22] Cerritelli F, Iacopini A, Galli M (2021). Evidence-based practice among Italian osteopaths: a national cross-sectional survey. BMC Complement Med Ther.

[23] Alvarez G, Justribo C, Sundberg T (2021). A national cross-sectional survey of the attitudes, skills and use of evidence-based practice amongst Spanish osteopaths. BMC Health Serv Res.

[24] Fleurence RL, Torgerson DJ (2004). Setting priorities for research. Health Policy.

[25] Heal C, Roberts G (2019). General practice research priority setting in Australia: Informing a research agenda to deliver best patient care. Aust J Gen Pract.

[26] Vaucher P (2023). PROCare Survey Material Prior to Data Collection. Version 1.2.

[27] Linstone HA, Turoff M (2011). Delphi: A brief look backward and forward. Technol Forecast Soc Change.

[28] Amorin-Woods LG, Woods BL, Moore CS (2022). Research Priorities of the Australian Chiropractic Profession: A Cross-Sectional Survey of Academics and Practitioners. J Manipulative Physiol Ther.

[29] Hubbard G, Grist F, Pope LM (2022). Survey to identify research priorities for primary care in Scotland during and following the COVID-19 pandemic. BMJ Open.

[30] Lee AD, deGraauw LC, Muir BJ (2021). A qualitative study investigating research priorities and investigative capacity in sports-focused chiropractic research, part 1 - identifying research priorities to inform a Delphi study. J Can Chiropr Assoc.

[31] O’Neill B, Aversa V, Rouleau K (2018). Identifying top 10 primary care research priorities from international stakeholders using a modified Delphi method. PLoS One.

[32] Synnot A, Bragge P, Lowe D (2018). Research priorities in health communication and participation: international survey of consumers and other stakeholders. BMJ Open.

[33] French SD, Beliveau PJH, Bruno P (2017). Research priorities of the Canadian chiropractic profession: a consensus study using a modified Delphi technique. Chiropr Man Therap.

[34] McKenna H, McDonough S, Keeney S (2014). Research priorities for the therapy professions in Northern Ireland and the Republic of Ireland: a comparison of findings from a Delphi consultation. J Allied Health.

[35] Kaur P, Chitra GA, Mehendale SM (2014). Perceptions of State Government stakeholders & researchers regarding public health research priorities in India: an exploratory survey. Indian J Med Res.

[36] Stevens KR, Ovretveit J (2013). Improvement research priorities: USA survey and expert consensus. Nurs Res Pract.

[37] Rankin G, Rushton A, Olver P (2012). Chartered Society of Physiotherapy’s identification of national research priorities for physiotherapy using a modified Delphi technique. Physiotherapy.

[38] Rushton A, Moore A (2010). International identification of research priorities for postgraduate theses in musculoskeletal physiotherapy using a modified Delphi technique. Man Ther.

[39] Bélanger M, Carpenter JG, Sabiston CM (2022). Identifying priorities for sport and physical activity research in Canada: an iterative priority-setting study. CMAJ Open.

[40] Nast I, Tal A, Schmid S (2016). Physiotherapy Research Priorities in Switzerland: Views of the Various Stakeholders. Physiother Res Int.

[41] Rubinstein SM, Bolton J, Webb AL (2014). The first research agenda for the chiropractic profession in Europe. Chiropr Man Therap.

[42] Bradley EH, Curry LA, Devers KJ (2007). Qualitative Data Analysis for Health Services Research: Developing Taxonomy, Themes, and Theory. Health Serv Res.

[43] Rampin R, Rampin V (2021). Taguette: open-source qualitative data analysis. JOSS.

[44] Thomson OP, Petty NJ, Moore AP (2013). Reconsidering the patient-centeredness of osteopathy. Int J Osteopath Med.

[45] Fahlgren E, Nima AA, Archer T (2015). Person-centered osteopathic practice: patients’ personality (body, mind, and soul) and health (ill-being and well-being). PeerJ.

[46] Steel A, Foley H, Redmond R (2020). Person-centred care and traditional philosophies in the evolution of osteopathic models and theoretical frameworks: Response to Esteves et al. Int J Osteopath Med.

[47] Keter D, Hutting N, Vogsland R (2024). Integrating Person-Centered Concepts and Modern Manual Therapy. JOSPT Open.

[48] Brewer MB, Gardner W (1996). Who is this “We”? Levels of collective identity and self representations. J Pers Soc Psychol.

[49] Braun V, Clarke V (2006). Using thematic analysis in psychology. Qual Res Psychol.

[50] Nylund KL, Asparouhov T, Muthén BO (2007). Deciding on the Number of Classes in Latent Class Analysis and Growth Mixture Modeling: A Monte Carlo Simulation Study. Struct Equ Modeling.

[51] Vaucher P, Draper-Rodi J, Carnes D (2025). PROCare-2023 data. Version 1. https://zenodo.org/doi/10.5281/zenodo.14826001.

[52] Andersen LN, Kristensen KL, Howell CM (2023). What matters to people with chronic musculoskeletal pain consulting general practice? Comparing research priorities across different sectors. Scand J Pain.

[53] Gill TK, Mittinty MM, March LM (2023). Global, regional, and national burden of other musculoskeletal disorders, 1990–2020, and projections to 2050: a systematic analysis of the Global Burden of Disease Study 2021. The Lancet Rheumatology.

[54] Oliveira CB, Ferreira GE, Buchbinder R (2023). Do national health priorities align with Global Burden of Disease estimates on disease burden? An analysis of national health plans and official governmental websites. Public Health (Fairfax).

[55] Pickering G, O’Keeffe M, Bannister K (2025). A pain research strategy for Europe: A European survey and position paper of the European Pain Federation EFIC. Eur J Pain.

[56] World Medical Association (2013). World Medical Association Declaration of Helsinki: ethical principles for medical research involving human subjects. JAMA.

