# Supplementary material for: European research Priorities for Osteopathic Care (PROCare): a sequential exploratory investigation and survey
Source: BMJ Open. 2025 Oct 16;15(10):e100757. doi: 10.1136/bmjopen-2025-100757 (PMC12530392; doi:10.1136/bmjopen-2025-100757)
Supplement: online supplemental file 1 [file bmjopen-15-10-s001.pdf]

# Supplementary file A

**Table A** – Population description; n (%)

| | BE<br>n=54 | BR<br>n=79 | CA<br>n=68 | CH<br>n=205 | DE<br>n=377 | ES<br>n=168 | FI<br>n=54 | FR<br>n=579 | IT<br>n=189 | UK<br>n=248 | Other<br>n=189 | All<br>n=2229 |
|---|---|---|---|---|---|---|---|---|---|---|---|---|
| Gender | | | | | | | | | | | | |
| Cisgender – women | 18 (33.3) | 45 (57.0) | 39 (57.3) | 117 (57.1) | 237 (62.9) | 66 (39.3) | 31 (57.4) | 269 (46.5) | 85 (45.0) | 148 (59.7) | 151 (42.3) | **1146 (51.4)** |
| Cisgender – men | 34 (63.1) | 28 (35.4) | 27 (38.7) | 79 (38.5) | 126 (33.4) | 97 (57.7) | 18 (33.3) | 292 (50.4) | 100 (52.9) | 86 (34.7) | 195 (54.6) | **993 (44.6)** |
| Transgender, Bigender, Agender | 0 (0) | 1 (1.3) | 1 (1.5) | 2 (1.0) | 4 (1.1) | 2 (1.2) | 2 (3.7) | 6 (1.0) | 1 (0.5) | 3 (1.2) | 5 (1.4) | **25 (1.1)** |
| Age (years) | | | | | | | | | | | | |
| < 35 | 8 (14.8) | 2 (2.5) | 13 (19.1) | 51 (24.9) | 62 (16.4) | 46 (27.4) | 16 (29.6) | 360 (62.2) | 94 (49.7) | 41 (16.5) | 87 (24.4) | **735 (33.0)** |
| 35 – 49 | 22 (40.7) | 38 (48.1) | 34 (50.0) | 97 (47.3) | 150 (39.8) | 92 (54.8) | 27 (50.0) | 173 (29.9) | 58 (30.7) | 63 (25.4) | 176 (49.3) | **847 (38.0)** |
| 50 – 64 | 16 (29.6) | 34 (43.0) | 19 (27.9) | 52 (25.4) | 137 (36.3) | 28 (16.7) | 7 (13.0) | 31 (5.3) | 31 (16.4) | 92 (37.1) | 83 (23.3) | **509 (22.8)** |
| ≥ 65 | 7 (13.0) | 5 (6.3) | 2 (2.9) | 1 (0.5) | 21 (5.6) | 1 (0.6) | 2 (3.7) | 12 (2.1) | 4 (2.1) | 44 (17.7) | 8 (2.2) | **106 (4.8)** |
| Interested parties | | | | | | | | | | | | |
| Patient | 2 (3.7) | 9 (11.4) | 6 (8.8) | 6 (2.9) | 19 (5.0) | 4 (2.4) | 9 (16.7) | 30 (5.2) | 8 (4.2) | 64 (25.8) | 7 (3.7) | **165 (7.4)** |
| Practitioner | 28 (51.8) | 20 (25.3) | 26 (38.2) | 136 (66.3) | 212 (48.7) | 38 (22.6) | 16 (29.6) | 217 (37.5) | 92 (48.7) | 78 (31.4) | 69 (36.5) | **939 (42.1)** |
| Student | 5 (9.3) | 12 (15.2) | 8 (11.8) | 14 (6.8) | 64 (17.0) | 48 (28.6) | 20 (37.0) | 133 (23.0) | 14 (7.4) | 40 (16.1) | 29 (15.3) | **389 (17.4)** |
| Educator | 5 (9.3) | 17 (21.5) | 4 (5.9) | 18 (8.9) | 30 (8.0) | 48 (28.6) | 5 (9.3) | 88 (15.2) | 20 (10.6) | 31 (12.5) | 34 (18.0) | **302 (13.5)** |
| Researcher | 3 (5.6) | 4 (5.1) | 8 (11.8) | 4 (1.9) | 11 (2.9) | 8 (4.8) | 1 (1.8) | 36 (6.2) | 9 (4.8) | 12 (4.8) | 12 (6.3) | **111 (5.0)** |
| Policy maker | 3 (5.6) | 5 (6.3) | 7 (10.3) | 2 (1.0) | 8 (2.1) | 10 (5.9) | 0 (0.0) | 15 (2.6) | 12 (6.3) | 13 (5.2) | 20 (10.6) | **95 (4.3)** |
| Preferred not to answer | 8 (14.8) | 12 (15.2) | 9 (13.2) | 25 (12.2) | 33 (8.7) | 12 (7.1) | 3 (5.6) | 60 (10.4) | 34 (18.0) | 10 (4.0) | 18 (9.5) | **228 (10.2)** |
| Profiles for setting priorities | | | | | | | | | | | | |
| Conservative | 30 (55.6) | 22 (27.8) | 28 (41.2) | 87 (42.4) | 164 (43.5) | 66 (39.3) | 31 (57.4) | 268 (46.3) | 74 (39.1) | 96 (38.7) | 87 (41.7) | **956 (42.9)** |
| Sceptic | 11 (20.4) | 5 (6.3) | 16 (23.5) | 58 (28.3) | 69 (18.3) | 22 (13.1) | 8 (14.8) | 140 (24.2) | 25 (13.2) | 66 (26.6) | 51 (14.3) | **451 (20.2)** |
| Enthusiast | 13 (24.1) | 52 (65.8) | 24 (35.3) | 60 (29.3) | 144 (38.2) | 80 (47.6) | 15 (27.8) | 171 (29.5) | 90 (47.6) | 86 (34.7) | 157 (44.0) | **822 (36.9)** |
| Estimated total number of osteopaths in country* | 1800 | 139 | 2937 | 1086 | 6612 | 9420 | 488 | 33000 | 13850 | 5439 | – | **–** |
| Response rate per 1000 osteopaths | 30 | 568 | 23 | 188 | 57 | 18 | 111 | 18 | 14 | 46 | – | **–** |

* as estimated in the 2020 OIA report [3]; BE = Belgium, BR = Brazil, CA = Canada, CH = Switzerland, DE = Germany, ES = Spain, FI = Finland, FR = France, IT = Italy, UK = United Kingdom
