# Supplementary material for: European research Priorities for Osteopathic Care (PROCare): a sequential exploratory investigation and survey
Source: BMJ Open. 2025 Oct 16;15(10):e100757. doi: 10.1136/bmjopen-2025-100757 (PMC12530392; doi:10.1136/bmjopen-2025-100757)
Supplement: online supplemental file 2 [file bmjopen-15-10-s002.pdf]

Supplementary file B

Figure B – Results from Confirmatory Factor Analysis of the Model for importance of research in osteopathic care (N=1,830).

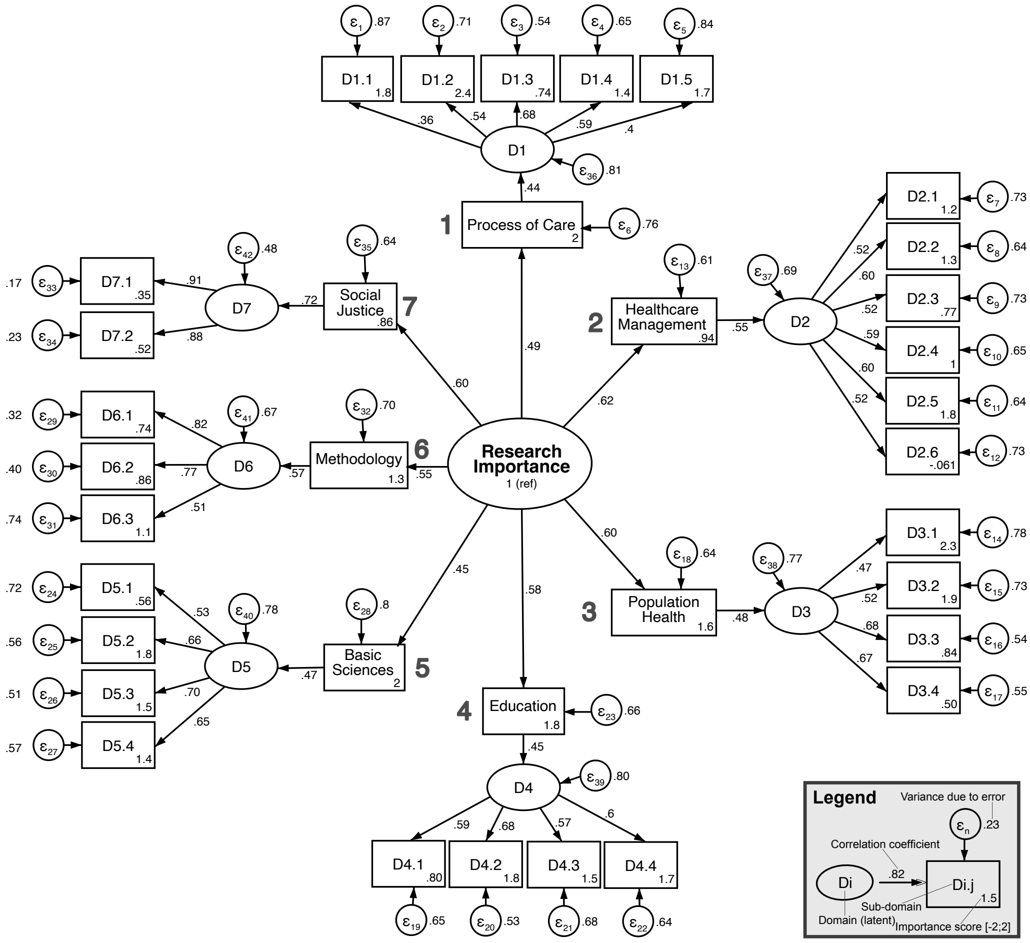

Domains and Sub-domains defining research importance are those constructed from the umbrella review and thematic analysis. Using their codes, they can be referred to specific domains of research in Figure 1, Tables 2 and Table 3.

Importance scores represent the average reported value of importance for each "Domain" and "Sub-Domain" provided by a 5-step Likert scale ranging from -2 (not important at all) to 2 (very important). Higher scores therefore represent "Domains" and "Sub-domains" to which participants accorded more importance.

Correlation coefficients represent the weight of each "Domain" or "Sub-Domain" in explaining the observed overall fluctuation of reported scores of importance of research between participants. Values range from zero to one. Higher values spots "Domains" and "Sub-Domains" that most influence participants overall affinity for research.
