# Supplementary material for: European research Priorities for Osteopathic Care (PROCare): a sequential exploratory investigation and survey
Source: BMJ Open. 2025 Oct 16;15(10):e100757. doi: 10.1136/bmjopen-2025-100757 (PMC12530392; doi:10.1136/bmjopen-2025-100757)
Supplement: Supplementary file 3 [file bmjopen-2025-100757.reviewer_comments.pdf]

PEER REVIEW HISTORY

BMJ Open publishes all reviews undertaken for accepted manuscripts. Reviewers are asked to complete a checklist review form and are provided with free text boxes to elaborate on their assessment. These free text comments are reproduced below.

ARTICLE DETAILS

Title (Provisional)

European Research Priorities for Osteopathic Care (PROCare): a sequential exploratory investigation and survey

Authors

Vaucher, Paul; Carnes, Dawn; Hohenschurz-Schmidt, David; Thomson, Oliver; Vogel, Steven; Arienti, Chiara; Bright, Philip; Bustins, Gerard; Esteves, Jorge; Koch Esteves, Nuno; Fawkes, Carol; Rinne, Sandra; Roura, Sonia; Treffel, Loïc; Wagner, Agathe; Draper-Rodi, Jerry

---

VERSION 1 - REVIEW

**Reviewer**	**1**
**Name**	**Stuber, Kent**
**Affiliation**	**Parker University, Research Center**
**Date**	**28-May-2025**
**COI**	**None**

I would like to thank the authors for submitting this manuscript. I would also like to thank the Editor for the opportunity to serve as a reviewer.

This manuscript, titled "European Research Priorities for Osteopathic Care

(PROCare): a mixed-methods investigation across stakeholders" represents an impressive international undertaking by this study team to identify research priorities for the Osteopathic profession in Europe. In general, the paper is well laid out and very well written. However, there are some issues that I have identified, and recommend that they be remedied before moving it further:

1. The term "stakeholder" (used throughout) is actually problematic in North America, as it evokes colonial implications. I understand that most of the authorship team is European based, and this may not have been considered. I would strongly encourage you to consider a term with less troubling history and consider "interested parties" or something similar.

2. It is a bit concerning that only a single researcher conducted the search, retrieval, etc. for the umbrella review. This should be cited as a limitation if nothing else.

3. ChatGPT 3.0 should be referenced appropriately.

4. The study should be explicitly reported using the Good Reporting of A Mixed Methods Study (GRAMMS) guideline.

5. I feel that there should be more explanation and referencing of the Latent Class Analysis as this leads to important findings within the Results section (identification of the 3 groups).

6. How would you describe this as a mixed methods design? I see you describe it as 'novel', and that is correct, but I wonder if perhaps it fits better as being described as either sequential exploratory or Convergent?

7. There is a general lack of description of the "mixing" required for a mixed methods study. How were the various different results integrated?

8. I wonder if the lack of input from American and Australian osteopathic communities (for example) might be considered a limitation?

9. The study conclusions are worded too strongly and should be toned down considerably. Phrasing as it introduces considerable bias to readers.

All in all, this is a unique study that should hopefully be illuminating for members of the osteopathic profession in Europe, while also potentially informing the approaches of other groups looking to set research priorities.

**Reviewer**	**2**
**Name**	**Wood, Lianne**
**Affiliation**	**University of Exeter**
**Date**	**21-Jul-2025**
**COI**	**None**

Well done on a comprehensive prioritisation process. This paper describes the methods used to define and prioritise the research priorities of European Osteopathic practitioners. The used a literature review, consensus methodology, survey development and then survey release with quantitative and qualitative analysis of survey results.

I have a few minor comments which may help to make the study more transparent.

Introduction:

1. You mention the difference between Osteopathic physicians in the USA, but do not really describe how Osteopaths may differ across Europe or what these differences may mean for the results. I wasn't sure really what the definition of Osteopaths in USA brings to this study other than to show variability.

Methods:

2. A flow chart may be helpful to demonstrate the different actions taken in underpinning this survey/ in describing participants who take part. A visual may be beneficial *but not absolutely necessary.

3. Studied population - It appears in line 16 that only UK patient populations were invited to participate in the survey. Is this why there was a majority UK respondents? It might be worth noting in the limitations section if this is indeed the case.

4. Would it be useful to provide a brief summary of the 16 studies and their research priorities identified in the literature review in the Appendix?

5. In section 4)Framework Finalisation and 5) development and validation. It is not clear who participated in this round. Was this the 19 osteopathic researchers? Would it be useful to add in the Appendix a baseline characterstics summary of these 19 that influences/ developed the framework in terms of contexts / perspective etc? It is tricky to disentangle whether the people in section 5 were the same as section 3 and or 4. Can you clarify please/ make it clear if the same core group went through all stages with the addition of extras/ if they were completely different groups.

6. PPI - Could you perhaps clarify here whether only UK patients and public were recruited or whether from other European countries as well?

**Reviewer**	**3**
**Name**	**Pohlman, Katherine A.**
**Affiliation**	**Parker University, Research Center**
**Date**	**30-Jul-2025**
**COI**	**None**

This manuscript presents a well-designed and thoughtfully executed investigation into research priorities within the osteopathic profession. The authors employed a mixed-methods approach combining qualitative framework development with a qualitative international cross-sectional survey to identify and analyze research priorities across the osteopathic profession. This study makes an important contribution to osteopathic research by providing a data-driven roadmap for future inquiry. Furthermore, it holds broader relevance by demonstrating a replicable approach to research priority-setting that can engage stakeholders, particularly research funders, in more informed and responsive ways. This is an exemplary piece of work that thoughtfully addresses a critical and often underexplored area of professional development. It reflects methodological rigor, stakeholder engagement, and forward-thinking implications. I fully support its publication and believe it will serve as a reference point for similar efforts in other health professions.

VERSION 1 - AUTHOR RESPONSE

Reviewer 1 - Kent Stuber

Comment R1-1: The term "stakeholder" (used throughout) is actually problematic in North America, as it evokes colonial implications. I understand that most of the authorship team is European based, and this may not have been considered. I would strongly encourage you to consider a term with less troubling history and consider "interested parties" or something similar.

Response R1-1: Thank you in helping us avoid using a term that could be offensive to some. Apparently, the sensitivity is also a UK issue as pointed out by Dr Caitlin Hafferty from Oxford University.

Action R1-1: We have changed the term to "interested parties" throughout the manuscript including Figures and Tables.

Comment R1-2: It is a bit concerning that only a single researcher conducted the search, retrieval, etc. for the umbrella review. This should be cited as a limitation if nothing else.

Response R1-2: While the umbrella review offers valuable insights, having conducted it by a single researcher introduces significant risks of bias and limitations. This includes increased subjectivity, limited perspective, and increased confirmation bias. There is little sound methodological research available to know which approach is best in reducing bias including the ideal number of independent analyst and ways to reach agreement when discrepencies occur. To avoid inconsistent data extraction, categorisation, and labelling, the entire process was initialy validated by five other researchers and then reviewed and revised during a workshop including 18 other experienced researchers with diverse expertise thereby assuring the quality, reliability, and validity of the findings from the umbrella review whilst sparing workload for each of them. Subjectivity and confirmation bias cannot however be entirely ruled out in the creation of the theoretical framework.

Action R1-2: The following statement was added to the limitation section within the discusion.
"While the umbrella review offers valuable insights, having conducted it by a single researcher introduces significant risks of bias and limitations. This was however mitigated by having 18 experienced researchers with diverse expertise fields in research review the entire process thoroughly. Nevertheless, subjectivity and confirmation bias cannot be entirely ruled out, and the theoretical framework should not be considered as entirely theory driven."

Comment R1-3: ChatGPT 3.0 should be referenced appropriately.

Response R1-3: This is now done.

Action R1-3: The following information was added: "OpenAI. (2023). *ChatGPT*-3.5 (May 3 version) [Large language model]. https://chat.openai.com/chat".

Comment R1-4: The study should be explicitly reported using the Good Reporting of A Mixed Methods Study (GRAMMS) guideline.

Response R1-4: Editors asked to also fill in the Cross checklist for surveys. As we decided to present the study as a “sequential exploratory” design, it seems unnecessary to also comlete the GRAMMS. Please let us know if you consider otherwise.

Action R1-4: Action: CROSS checklist added to the submitted documents.

Comment R1-5: I feel that there should be more explanation and referencing of the Latent Class Analysis as this leads to important findings within the Results section (identification of the 3 groups).

Comment R1-5: Response: The statistical methods section already states the following: “Latent class analysis was conducted using maximum likelihood estimation to identify distinct profiles in priority-setting approaches. Model selection compared solutions with 2-7 classes using Bayesian Information Criterion (BIC) as the primary fit index, supplemented by Akaike Information Criterion (AIC) when BIC showed unstable fluctuation. The optimal solution was determined by lowest BIC value combined with theoretical interpretability of the resulting classes.” The full command and automated output from the statistical analysis is made available publicly on a registry (https://zenodo.org/records/14826002). The detailed methods are provided in section 3.9, Patterns for setting priorities using LCA, line 1569.

Action R1-5: The reference for class number determination was added.

Comment R1-6: How would you describe this as a mixed methods design? I see you describe it as 'novel', and that is correct, but I wonder if perhaps it fits better as being described as either sequential exploratory or Convergent?

Response R1-6: “Sequential exploratory approach” indeed seems to better describe what was done.

Action R1-6: References to a mixed-methods approach were deleted.

Comment R1-7. There is a general lack of description of the "mixing" required for a mixed methods study. How were the various different results integrated?

Response R1-7: The methods was described as a Sequential exploratory design. Given each step is indispendible for the next, it seems straightforward on how each step added towards the final measuring instrument included in the survey.

Action R1-7: The term Sequential exploratory design was used instead of Mixed.

Comment R1-8. I wonder if the lack of input from American and Australian osteopathic communities (for example) might be considered a limitation?

Response R1-8: The limitation of running the survey with countries affiliated to Osteopathy Europe indeed needs to be addressed.

Australia has a very similar scope of practice for osteopathic care as the UK and Switzerland which are largly represented. Australia also recently ran a survey on the matter which suggests their position is quite similar to the rest of the world.

The USA situation seems more complex. There are important differences in scope of practice between practitioners from USA and other countries in the world. The considerable professional differences makes US and non-US osteopathy essentially incomparable. Running such a survey in the USA would indeed be of interest.

Action R1-8: Added limitation for USA.

Comment R1-G: The study conclusions are worded too strongly and should be toned down considerably. Phrasing as it introduces considerable bias to readers.

Response R1-9: The conclusion is indeed worded inappropriatly for a scientific paper. It was rewritten in consequence.

Action R1-9: The entire conclusion was reworded.

Reviewer: 2 - Lianne Wood

Comment R2-1: You mention the difference between Osteopathic physicians in the USA, but do not really describe how Osteopaths may differ across Europe or what these differences may mean for the results. I wasn't sure really what the definition of Osteopaths in USA brings to this study other than to show variability.

Response R2-1: For practical reasons, this study mainly focuses on Europe and state members from Osteopathy Europe (Canada and Brazil). In Europe, standards for practicing as an osteopath vary between countries ranging from countries with no regulation whatsoever (ex. Germany, Netherlands, Spain) to those practicing with regulated university degree primary care status (ex. UK, Switzerland). Up to 2020, one osteopath out of two in the world was trained in the US. It therefore seemed important to highlight this difference and justify the usefulness of studying European settings.

Action R2-1: The following sentence was added to the introduction: "In Europe, even if unified standards for education have been formulated [3], osteopathic care and its integration in health systems varies considerably between countries ranging from countries with no regulation (e.g. Germany, Netherlands, Spain), passing by countries with regulation as complementary medicine without medical scope (e.g. France, Italy), to those practicing with regulated university degree primary care status (e.g. UK, Switzerland) [4].

Comment R2-2: A flow chart may be helpful to demonstrate the different actions taken in underpinning this survey/ in describing participants who take part. A visual may be beneficial *but not absolutely necessary.

Response R2-2: Indeed, a flow chart showing the different sequential step of the study would be useful. However, we are limited to four Figures and had to make some choices. Visuals for reporting results seem essential and our choices went for

priviledging results rather than methods.

Comment R2-3: Studied population - It appears in line 16 that only UK patient populations were invited to participate in the survey. Is this why there was a majority UK respondents? It might be worth noting in the limitations section if this is indeed the case.

Response R2-3: Patient participants were indeed only included from the UK contributing to the higher number of responders. This is indeed an important limitation that was added to the limitation section.

Action R2-3: The following statement was added to the limitation section: "Some countries (i.e. USA, Australia) or interested parties (i.e. patients) are underrepresented."

Comment R2-4: Would it be useful to provide a brief summary of the 16 studies and their research priorities identified in the literature review in the Appendix?

Response R2-4: Giving additional details to how the qualitative thematic analysis was done is important. These details are already accessible and referenced in the Data sharing section (https://zenodo.org/records/8322740) as the document number 6.

Action R2-4: We have added the link to the document in the methods section.

Comment R2-5: In section 4)Framework Finalisation and 5) development and validation. It is not clear who participated in this round. Was this the 1G osteopathic researchers? Would it be useful to add in the Appendix a baseline characterstics summary of these 1G that influences/ developed the framework in terms of contexts / perspective etc? It is tricky to disentangle whether the people in section 5 were the same as section 3 and or 4. Can you clarify please/ make it clear if the same core group went through all stages with the addition of extras/ if they were completely different groups.

Response R2-5: Indeed, the group of 19 included the 5 researchers implicated in the prior steps. This was clarified.

Action R2-5: Details were added on who participated to each phase.

Comment R2-6: PPI - Could you perhaps clarify here whether only UK patients and public were recruited or whether from other European countries as well?

Response R2-6: Indeed, given we only had access to the UK official health representative group, no other patient group were included in the survey itself. However, for the evaluation of the survey itself, patients from a variety of countries were included (i.e. UK, Switzerland).

Action R2-6: The origin of participants was added to the methods section.

Reviewer 3 - Katherine A. Pohlman

No comments were made that needed a response.

VERSION 2 - REVIEW

**Reviewer**	**1**
**Name**	**Stuber, Kent**
**Affiliation**	**Parker University, Research Center**
**Date**	**01-Sep-2025**
**COI**	

My sincere thanks to the authors for their responses to my previous review. I feel that they have generally addressed my previous concerns quite well.

There are two final comments that I'll make:

1. 'Sequential exploratory' is a type of mixed method study design. So, removing reference to mixed methods throughout the manuscript isn't really appropriate.

2. I would also like to see maybe a couple of sentences in reference to integration of your qualitative and quantitative findings accordingly, as in how did the things learned from one stage of the study inform what you did in the next stage? However, I'll leave this to the Editor's discretion.

Otherwise, I think this paper is important for the osteopathic profession and could be informative for other professions as well. Well done!

VERSION 2 - AUTHOR RESPONSE

Reviewer 1 - Kent Stuber

Comment R1-1. 'Sequential exploratory' is a type of mixed method study design. So, removing reference to mixed methods throughout the manuscript isn't really appropriate.

Response R1-1: We have revised the document to add this precision.

Action R1-1: The term “mixed methods” was reintroduced in the abstract and the method sections.

Comment R1-2. I would also like to see maybe a couple of sentences in reference to integration of your qualitative and quantitative findings accordingly, as in how did the things learned from one stage of the study inform

what you did in the next stage? However, I'll leave this to the Editor's discretion.

Response R1-2: We made the sequential approach clearer.

Action R1-2: The following paragraph replaced the first section of the design description: "The PROCare framework was developed using a sequential exploratory mixed-methods design, where findings from each phase informed the next. The umbrella review generated a master list of potential priorities, which was thematically analysed to produce a taxonomy of domains, subdomains, and topics. This taxonomy was refined through expert validation, resulting in a conceptual framework that guided the construction of the survey. The draft survey was then tested, validated, and culturally adapted before being deployed between August and October 2023 to quantitatively assess the perceived importance of research priorities among practitioners, educators, researchers, policymakers, and from the public including patients."
